# Transcriptomic Profile of New Gene Markers Encoding Proteins Responsible for Structure of Porcine Ovarian Granulosa Cells

**DOI:** 10.3390/biology10111214

**Published:** 2021-11-20

**Authors:** Jakub Kulus, Magdalena Kulus, Wiesława Kranc, Karol Jopek, Maciej Zdun, Małgorzata Józkowiak, Jędrzej M. Jaśkowski, Hanna Piotrowska-Kempisty, Dorota Bukowska, Paweł Antosik, Paul Mozdziak, Bartosz Kempisty

**Affiliations:** 1 Department of Diagnostics and Clinical Sciences, Institute of Veterinary Medicine, Nicolaus Copernicus University in Torun, 87-100 Torun, Poland; jakub.kulus@umk.pl (J.K.); jmjaskowski@umk.pl (J.M.J.); dbukowska@umk.pl (D.B.); 2 Department of Veterinary Surgery, Institute of Veterinary Medicine, Nicolaus Copernicus University in Torun, 87-100 Torun, Poland; magdalena.kulus@umk.pl (M.K.); pantosik@umk.pl (P.A.); 3 Department of Anatomy, Poznan University of Medical Sciences, 60-781 Poznan, Poland; wkranc@ump.edu.pl; 4 Department of Histology and Embryology, Poznan University of Medical Sciences, 60-781 Poznan, Poland; kjopek@ump.edu.pl; 5 Department of Basic and Preclinical Sciences, Institute of Veterinary Medicine, Nicolaus Copernicus University in Torun, 87-100 Torun, Poland; maciejzdun@umk.pl (M.Z.); hpiotrow@ump.edu.pl (H.P.-K.); 6 Department of Toxicology, Poznan University of Medical Sciences, 61-631 Poznan, Poland; 71389@student.ump.edu.pl; 7 Prestage Department of Poultry Science, College of Agriculture and Life Sciences, North Carolina State University, Raleigh, NC 27695, USA; pemozdzi@ncsu.edu

**Keywords:** porcine granulosa cells, extracellular matrix, gene expression profile, folliculogenesis, reproductive physiology

## Abstract

**Simple Summary:**

The extracellular matrix (ECM) is involved in many physiological processes that occur in the ovary and affect reproduction in animals and humans. The ECM has been shown to significantly affect folliculogenesis, ovulation, and corpus luteum formation. This is mainly due to the involvement of ECM in intercellular signaling. In the present study, we report the gene expression profile of porcine granulosa cells during their primary in vitro culture. The genes presented are related to ECM formation but also to cadherins and integrins that influence intercellular dialogue. During the study, it was shown that most of the genes were upregulated. A detailed understanding of the expression of genes such as POSTN, CHI3L1, CAV-1, IRS1, DCN in in vitro culture of granulosa cells may provide a basis for further studies on the molecular mechanisms occurring within the ovary. Knowledge of ECM-related gene expression within granulosa cells can also be used to study the recently discovered stemness of these cells. Moreover, the presented data may serve for the development of assisted reproduction techniques, which, especially in vitro, are becoming increasingly common.

**Abstract:**

The extracellular matrix (ECM) in granulosa cells is functionally very important, and it is involved in many processes related to ovarian follicle growth and ovulation. The aim of this study was to describe the expression profile of genes within granulosa cells that are associated with extracellular matrix formation, intercellular signaling, and cell–cell fusion. The material for this study was ovaries of sexually mature pigs obtained from a commercial slaughterhouse. Laboratory-derived granulosa cells (GCs) from ovarian follicles were cultured in a primary in vitro culture model. The extracted genetic material (0, 48, 96, and 144 h) were subjected to microarray expression analysis. Among 81 genes, 66 showed increased expression and only 15 showed decreased expression were assigned to 7 gene ontology groups “extracellular matrix binding”, “extracellular matrix structural constituent”, “binding, bridging”, “cadherin binding”, “cell adhesion molecule binding”, “collagen binding” and “cadherin binding involved in cell-cell adhesion”. The 10 genes with the highest expression (POSTN, ITGA2, FN1, LAMB1, ITGB3, CHI3L1, PCOLCE2, CAV1, DCN, COL14A1) and 10 of the most down-regulated (SPP1, IRS1, CNTLN, TMPO, PAICS, ANK2, ADAM23, ABI3BP, DNAJB1, IGF1) were selected for further analysis. The results were validated by RT-qPCR. The current results may serve as preliminary data for further analyses using in vitro granulosa cell cultures in assisted reproduction technologies, studies of pathological processes in the ovary as well as in the use of the stemness potential of GCs.

## 1. Introduction

The extracellular matrix (ECM) is an extremely important structure present in all tissues of the animal organism. It exhibits various functions and is composed of many substances of different origins [1,2]. The major components of the extracellular matrix include two groups of molecules. The first is the fibrous proteins, which include collagens, elastin, fibronectin, and laminin. The most common of these proteins is collagen, which provides a support for tissue scaffolding but also affects cell adhesion, cell migration, and chemotaxis [3]. The second group are proteoglycans, which consist of a protein core to which glycosaminoglycans are attached. These molecules are highly hydrophilic, causing the formation of a hydrogel that fills the space between the fibrous proteins of the ECM. Additionally, proteoglycans are involved in many signaling pathways related to insulin-like growth factor 1 receptor (IGFIR), epidermal growth factor receptor (EGFR), low-density lipoprotein-receptor-related protein 1 (LRP1) [3]. The fact that an extracellular matrix is composed of a variety of components results in a vast array of functions [4]. It is a unique microenvironment for cells (including the delivery of osmotic forces), a mechanical support or pathway for the passage of nutrients, hormones and extracellular signals to the target cells as well as regulation of gene expression and cytokine release [1,5]. The complicated ultrastructure of the matrix provides the filter material, and the ability to bind growth factors required for local action. The ECM also influences cell behavior by participating in the migration, anchoring, division, and death of cells. Taking into account all these functions, it can be stated that it enables cells to specialize in a specific way by creating their microenvironment [6]. The role of the extracellular matrix in the development of cancer has also been demonstrated, including those associated with epithelial cells in the ovary [7]. The role of the ECM has also been described in the pathogenesis of other diseases related to the reproductive system, including PCOS (polycystic ovary syndrome) [8,9,10] and POI (premature ovarian insufficiency) [11,12]. The extensively described involvement of extracellular matrix components in signaling pathways within the ovary highlights the role of the ECM in folliculogenesis [13] and in pathological processes [14] which altogether may provide valuable therapeutic insights. Until now, little is known about the molecular-level processes involved in the ECM and its role in the maturation and growth of mammalian ovarian follicles, and this role seems to be significant [15]. An example is the presence of different laminin chains in ovarian follicles depending on their developmental stage, which is associated with antrum formation [16]. In addition, the expression of genes related to ECM remodeling was also found to change during follicle maturation [17]. ECM in ovarian follicles is expressed in various compartments, such as basal lamina, follicular fluid, zona pellucida, granulosa membrane, or cumulus [6]. The basal lamina is a flat sheet of extracellular matrix, which is specialized in separating the epithelial cells lying on it from the lower layers. Its structure is of a lattice type consisting of a network of type IV collagen and laminine. Entaxins/nidogen are attached to this network, stabilizing the structure. In various proportions fibronectin, heparan sulfate, and others are also connected [6,18,19,20]. Furthermore, these components may be present in different conforms, composed of various chains, thus affecting the uniqueness of a specific basal lamina, which in turn affects its functionality [6,21]. 

It is important to consider the role of cell signaling on reproductive efficiency, including via the ECM, cadherins, and integrins [22,23,24,25,26]. Cadherins and integrins are among the transmembrane proteins that are closely involved in intercellular signaling. The former are responsible for cell-to-cell signaling and their action is closely related to catenins (α, β and p120). The second are responsible for signaling between the cell and the ECM. The action of both groups of proteins is through their effect on F-actin [26]. Several groups are distinguished among the cadherins: N-cadherin (neural cadherin), P-cadherin (placental cadherin), R-cadherin (retinal cadherin), VE-cadherin (vascular endothelial cadherin), and the E-cadherin (epithelial cadherin) found in the epithelium of various tissues and have been implicated in the function of the reproductive system [22]. More than two decades ago, the expression of these transmembrane proteins in porcine GCs was described and linked to the development of ovarian follicles by maintaining the structural integrity of the follicle through E-cadherin [27]. Furthermore, a positive correlation between e-cadherin expression and the pool of primary follicles in the mouse ovary formed during embryonic development was demonstrated due to effects on cell–cell junction integrity and NOVOX expression [28] which may affect the occurrence of POI. These proteins also affect the fertilization process by allowing the interaction between sperm and oviduct epithelium and oocyte which was confirmed in a bovine model [29]. Intensive research in recent years on granulosa cells has further demonstrated their stemness potential. To date, human-derived granulosa cells have demonstrated the ability to differentiate into muscle, cartilage, bone, and even nerve tissue [30,31,32,33]. Porcine granulosa cells have shown the ability to differentiate into bone tissue [34,35]. These data suggest the possibility of using these cells in the treatment of degenerative diseases and also in regenerative medicine. Further studies conducted on porcine GCs, considered as an animal model for humans, may provide important information in the context of using these cells in therapy. Granulosa cells play an important role in the processes of folliculogenesis and oogenesis [36,37]. One of the most important functions of GCs is their participation in steroidogenesis as they perform aromatization of androgens to estrogens, which are secreted into the follicular fluid. In addition, the proliferation of GCs leads to the growth and development of the entire ovarian follicle [38]. The aim of this study was to determine the expression profile of genes responsible for processes related to cadherin and collagen binding and structuralization of ECM in porcine granulosa cells cultured in vitro. The present study compared the expression levels of selected ECM-related genes in vivo (0 h) and during in vitro culture. Cells isolated from the natural microenvironment (which for GCs is the ovarian follicle) and subjected to in vitro culture show an altered gene expression profile. Therefore, the mechanisms governing expression profile dynamics may be the basis for further research, especially in the field of assisted reproduction techniques based on in vitro culture. Additionally, characterization of extracellular matrix-related genes within them may elucidate the molecular basis of GCs function, which is important to elucidate the pathogenesis of ovarian-related diseases. The results obtained in this study, show significant upregulation of expression levels of genes related to both ECM formation and function, confirm the occurrence of these processes in vitro during GCs culture, which is associated with the development of these cells. The expression patterns observed in the current study, together with the positive effects of ECM on the efficiency of cell differentiation reported in the literature [39,40], may provide a valuable background for further research in studies on the pluripotency of GCs [41,42]. 

## 2. Materials and Methods

### 2.1. Animals

A total of 40 crossbred Landrace gilts with a median age of 170 days and weight of 98 kg were used in this study. All animals were housed under identical conditions. The animals in the study reached sexual maturity as pigs become sexually mature at 4–6 months of age. 

### 2.2. Collection of Porcine Ovarian Granulosa Cells

Ovaries (*n* = 80) were recovered at slaughter and transported to the laboratory at 38 °C in 0.9% NaCl within 30 min. In the laboratory, the ovaries of each animal were placed in PBS supplemented with fetal bovine serum (FBS; Sigma-Aldrich Co., St. Louis, MO, USA). Thereafter, single preovulatory large follicles, with a diameter estimated greater than 5 mm (*n* = 300), were opened into a sterile Petri dish by puncturing using a 5 ml syringe and 20 G needle, and the cumulus-oocyte complexes (COCs) and follicular fluid (FF) were recovered. The transcriptomic profile of mural GCs, which constitute a significant majority among the GCs population was analyzed. The follicular fluid was used to isolate GCs, whereas the COCs were discarded. The extracted follicular fluid after discarding COCs was filtered through sterile nylon cell strainers with a mesh diameter of 40 µm (Biologix Group, Shandong, China) to eliminate tissue debris and larger cell aggregates (including blood cells) or epithelium. The resulting suspension was centrifuged at room temperature for 10 min, 200 rpm, to obtain individual cell fractions. The GCs pellet was then resuspended in collagenase type I solution (Gibco, Thermo-Fischer Scientific, Waltham, MA, USA) 1 mg/1 mL DMEM and incubated 10 min in a 37 °C water bath and centrifuged (under the same conditions). The obtained cell pellet was resuspended in culture medium to establish in vitro culture under the conditions described below. Granulosa cells collected from ovarian follicles were pooled to homogenize the sample.

### 2.3. In Vitro Primary Culture of Porcine Granulosa Cells

A primary in vitro culture model was used in this study with four time intervals for each biological repeat. For microarray expressions, cultures were maintained in two biological replicates for each time interval. For validation by RT-qPCR, cultures were maintained in a triplicate biological sample model for each time interval. Primary cultures were established from GCs in four bottles with 3 × 10^6^ cells per dish (25 cm^2^ cell culture flask, TPP, Trasadingen, Switzerland). The number of cells and their viability were assessed using the ADAM Automatic Cell Counter (NanoEnTek, Waltham, MA, USA). From the cell suspension, a 20 µL sample for number and viability analysis was stained with propium iodide and examined in a fluorescence analyzer on disposable microchips. By staining the cell nuclei, the counter is able to distinguish single cells in aggregates. Only those samples with viability above 85% were used for further studies. Cells in culture were kept until culture termination when the material was collected at 0 h, 48 h, 96 h, 144 h. The culture medium was changed every 72 h. 

**Figure 1:**
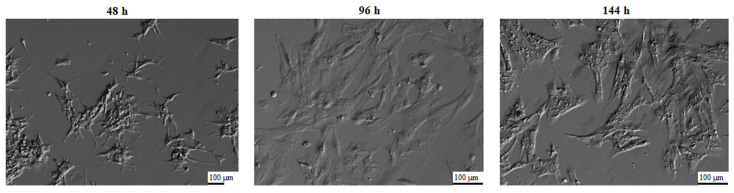
Porcine granulosa cells cultures used for the experiment, presenting changes in their morphology, following 48 h, 96 h, and 144 h of in vitro culture (magnification ×100 (48 h), magnification ×200 (96 h, 144 h)).

Culture medium consisted of Dulbecco’s Modified Eagle’s Medium (DMEM, Sigma-Aldrich, Saint Louis, MO, USA), 2% fetal calf serum (FCS) (PAA, Linz, Austria), 10 mg/mL ascorbic acid (Sigma-Aldrich, Saint Louis, MO, USA), 0.05 μM dexamethasone (Sigma-Aldrich, Saint Louis, MO, USA), 200 mM L-glutamine (Invitrogen, Carlsbad, CA, USA), 10 mg/mL gentamycin (Invitrogen, Carlsbad, CA, USA), 10,000 units/mL penicillin and 10,000 μg/mL streptomycin (Invitrogen, Carlsbad, CA, USA). Cells were cultivated at 38.5 °C under aerobic conditions (5% CO_2_). Once the adherent cells were more than 80% confluent, they were detached with 0.05% trypsin-EDTA (Invitrogen, Carlsbad, CA, USA) for 3 min. and then passaged. The morphology of the GCs was evaluated using an inverted phase-contrast microscope, and the results of these observations are presented in Figure 1. GCs underwent significant morphological changes. When the cells were seeded into culture bottles, the shape of the cells was close to spherical, where the cells formed a suspension in the medium. After 24 h of culture, the cells became adherent to the medium, and after 48 h, the cells assumed a star-like shape. At subsequent time intervals, the GCs became wider, more fibroblast-like. The strong adherence to the dish surface, shape change, and flattening of the cells is related to the secretion of extracellular matrix components, which correlates with the increased expression of ECM-related genes during the study.

### 2.4. Microarray Expression Analysis and Statistics

The Affymetrix procedure was previously described by Trejter et al. [43] and used in studies involving porcine oviduct epithelial cells (OECs) [44,45,46] as well as oocytes [47,48,49]. Briefly, cDNA was subjected to Total RNA (100 ng) (Ambion^®^ WT Expression Kit, Thermo Fisher Scientific Inc., Wilmington, DE, USA). Obtained cDNA was biotin labeled and fragmented by Affymetrix GeneChip^®^ WT Terminal Labeling and Hybridization (Affymetrix, Santa Clara, CA, USA). Biotin-labeled fragments of cDNA (5.5 μg) were hybridized to Affymetrix^®^ Porcine Gene 1.1 ST Array Strip (Affymetrix, Santa Clara, CA, USA) (48 °C/20 h). Then, microarrays were washed and stained according to the technical protocol using Affymetrix GeneAtlas Fluidics Station (Affymetrix, Santa Clara, CA, USA). Subsequently, the array strips were scanned by the Imaging Station of the GeneAtlas System (Affymetrix, Santa Clara, CA, USA). The preliminary analysis of the scanned chips was performed using Affymetrix GeneAtlasTM Operating Software. The quality of gene expression data was checked according to quality control criteria provided by the software. Obtained CEL files were imported into the downstream data analysis software. All of the presented analyses and graphs were performed by Bioconductor and R programming language. Each CEL file was merged with a description file. In order to correct background, normalize and summarize results, we used Robust Multiarray Averaging (RMA) algorithm. 

Statistical significance of analyzed genes was performed by moderated t-statistics from the empirical Bayes method. Obtained *p*-value was corrected for multiple comparisons using Benjamini and Hochberg’s false discovery rate. The selection of significantly changed gene expression was based on a *p*-value under 0.05 and expression fold higher than 2. Differentially expressed genes were subjected to the selection of genes involved in cadherin and collagen binding and structuralization of extracellular matrix components. Differentially expressed gene lists were uploaded to DAVID software (Database for Annotation, Visualization and Integrated Discovery), where “extracellular matrix binding”, “extracellular matrix structural constituent”, “binding, bridging”, “cadherin binding”, “cell adhesion molecule binding”, “collagen binding” and “cadherin binding involved in cell-cell adhesion” GO MF terms were obtained. Expression data of these genes were subjected to a hierarchical clusterization procedure and presented as a heatmap graph. Detailed analysis of genes belonging to selected GO MF terms was presented as plots using “GOplot” library [50] and “ClusterProfiler” R package [51]. In the chosen gene sets, we investigated their mutual relations using the GOplot package. Moreover, the GOplot package was used to calculate the z-score (difference in the number of up- and down-regulated genes divided by the square root of the count). The z-score analysis allowed us to compare the enrichment of the selected GO BP terms. 

Using STRING10 (Search Tool for the Retrieval of Interacting Genes, STRING Consortium, Lausanne, Switzerland) software, interactions between genes and the proteins they encode have been investigated. The STRING database includes information on protein/gene interactions, including experimental data, computational prediction methods, and public text collections. The STRING database engine supplied the molecular network of interactions formed between the chosen genes. Search criteria are provided based on gene/protein co-occurrence in scientific texts (textmining), coexpression, and experimentally observed interactions. 

The functional interaction between genes that belong to the chosen GO BP terms was investigated by the REACTOME FIViz application to the Cytoscape 3.8.2 software (San Diego, CA, USA). The Reactome FIViz app is designed to find pathways and network patterns related to cancer and other types of diseases. This app accesses the pathways stored in the Reactome database, allowing to do pathway enrichment analysis for a set of genes, visualize hit pathways using manually laid-out pathway diagrams directly in Cytoscape, and investigate functional relationships among genes in hit pathways. The app can also access the Reactome Functional Interaction (FI) network, a highly reliable, manually curated pathway-based protein functional interaction network covering over 60% of human proteins. 

### 2.5. Real-Time Quantitative Polymerase Chain Reaction (RT-qPCR) Analysis

Total RNA was isolated from GCs in 0 h and after 48 h, 96 h, and 144 h in vitro culture using an RNeasy mini column from Qiagen GmbH (Qiagen GmbH, Hilden, Germany). The RNA samples were resuspended in 20 µl of RNase-free water and stored in liquid nitrogen. RNA samples were treated with DNase I and reverse-transcribed (RT) into cDNA. RT-qPCR was conducted in a LightCycler real-time PCR detection system (Roche Diagnostics GmbH, Mannheim, Germany) using SYBR^®^ Green I (Master Mix Qiagen GmbH, Hilden, Germany) as a detection dye, and target cDNA was quantified using the relative quantification method. The relative abundance of analyzed transcripts in each sample was standardized to the internal standard glyceraldehyde-3-phosphate dehydrogenase (GAPDH). For amplification, 2 µL of cDNA solution was added to 18 µL of QuantiTect^®^ SYBR^®^ Green PCR (Master Mix Qiagen GmbH, Hilden, Germany) and primers (Table 1). One RNA sample of each preparation was processed without the RT reaction to provide a negative control for subsequent PCR.

To quantify the specific genes expressed in the GCs, the expression levels of specific mRNAs in each sample were calculated relative to PBGD and ACTB. To ensure the integrity of these results, the additional housekeeping gene 18S was used as an internal standard to demonstrate that PBGD and ACTB mRNAs were not differentially regulated in GC groups. The gene for 18S rRNA expression has been identified as an appropriate housekeeping gene for use in quantitative PCR studies. Expression of PBGD, ACTB, and 18S mRNA was measured in cDNA samples from isolated GCs. The statistical significance of the analyzed genes was performed using moderated t-statistics from the empirical Bayes method. The obtained *p*-value was corrected for multiple comparisons using Benjamini and Hochberg’s false discovery rate.

## 3. Results

**Figure 2:**
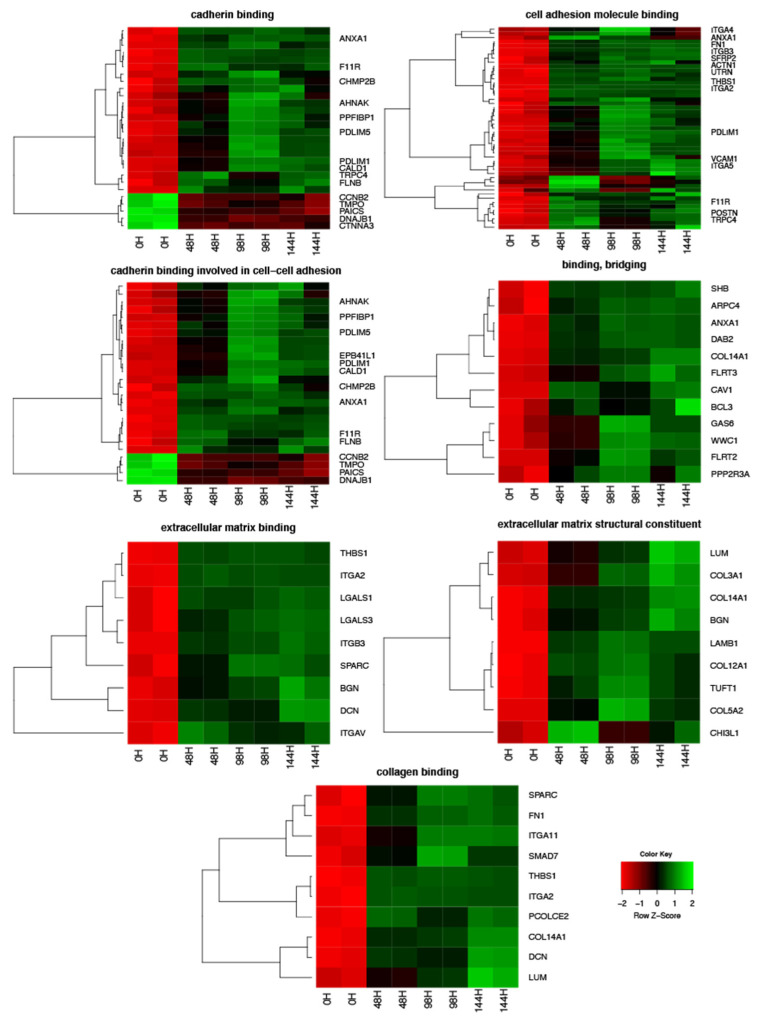
Heatmaps presenting differentially expressed genes involved in “extracellular matrix binding”, “extracellular matrix structural constituent”, “binding, bridging”, “cadherin binding”, “cell adhesion molecule binding”, “collagen binding” and “cadherin binding involved in cell-cell adhesion” based on GO MF terms. Each row on the *Y*-axis represents a single transcript. The red color indicates downregulated genes while the green indicates upregulated.

The Affymetrix^®^ Porcine Gene 1.1 ST Array Strip (Affymetrix, Santa Clara, CA, USA) for the microarray gene expression analysis of porcine granulosa cells allows the study of the gene expression of 27,558 transcripts at 0, 48, 96, and 144 h of in vitro granulosa cell culture. Genes with more than 2-fold changes and corrected *p*-values less than 0.05 were selected for downstream analysis. A total of 3380 differentially expressed genes (DEGs) were identified according to the above criteria. The list of DEGs was uploaded to the GEO database (ID:GSE134361). Microarray gene expression analysis with subjecting the list of DEGs to DAVID software, which showed that the genes can be assigned to 1901 GO BP (Gene Ontology Biological Process), 162 GO MF (Gene Ontology Molecular Function), and 182 GO CC (Gene Ontology Cellular Component) terms. Gene Ontology is the knowledge database of gene function and provides the basis for computational analyses used in molecular biology and genetics. This paper focused on the genes involved in cadherin and collagen binding and structuralization of extracellular matrix components. The DAVID software indicated the following GO MF terms, which cover the above processes: “extracellular matrix binding”, “extracellular matrix structural constituent”, “binding, bridging”, “cadherin binding”, “cell adhesion molecule binding”, “collagen binding” and “cadherin binding involved in cell-cell adhesion”. The 81 genes involved in those processes were clustered using hierarchical clustering and presented as heatmaps (Figure 2). Interestingly, 66 genes were upregulated, which is the greater part of the list of genes used for hierarchical clustering. Only 15 genes, in contrast, were downregulated. Some of the downregulated genes form small clusters in “cadherin binding” and “cadherin binding involved in cell-cell adhesion” GO MF terms. The direction of expression change (upregulation or downregulation) was maintained in granulosa cell culture in subsequent points of analysis (after 48, 96, and 144 h of in vitro culture). The mean value of the fold change ratio of each gene between 48 h, 96 h, and 144 h was calculated. For further analysis we choose the 10 most significantly upregulated and downregulated genes and their symbols, fold changes, and corrected *p*-values are shown in Table 2.

**Figure 3:**
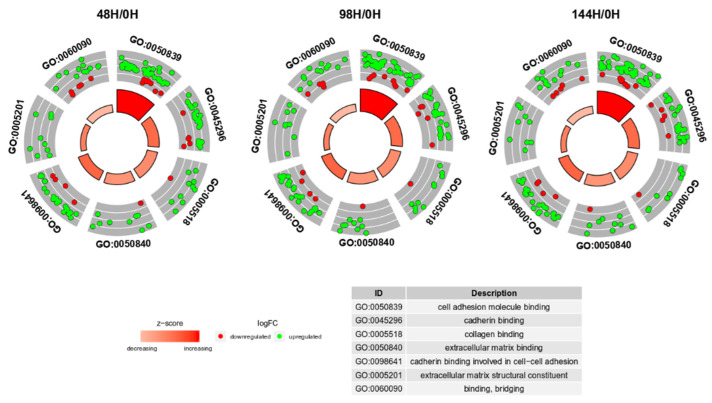
The circular scatter plots of differentially expressed genes involved in “extracellular matrix binding”, “extracellular matrix structural constituent”, “binding, bridging”, “cadherin binding”, “cell adhesion molecule binding”, “collagen binding” and “cadherin binding involved in cell-cell adhesion” GO MF terms. Each dot represents a single gene. The z-scores were presented as segments of inner circles.

Z-scores, reveal whether the molecular function is more likely to be decreased (negative value) or increased (positive value). The z-scores were presented as segments of inner circles in Figure 3. As can be seen from the figure, the expression of most genes was increased (green dots) in all ontological groups. The z-scores had positive values, indicating that the GO MF terms were upregulated. The “cell adhesion molecule binding” term contained the highest number of genes. The expression pattern did not change at any of the analyzed time points. Considering all information, the subsequent analysis was based only on 48H/0H comparison.

**Figure 4:**
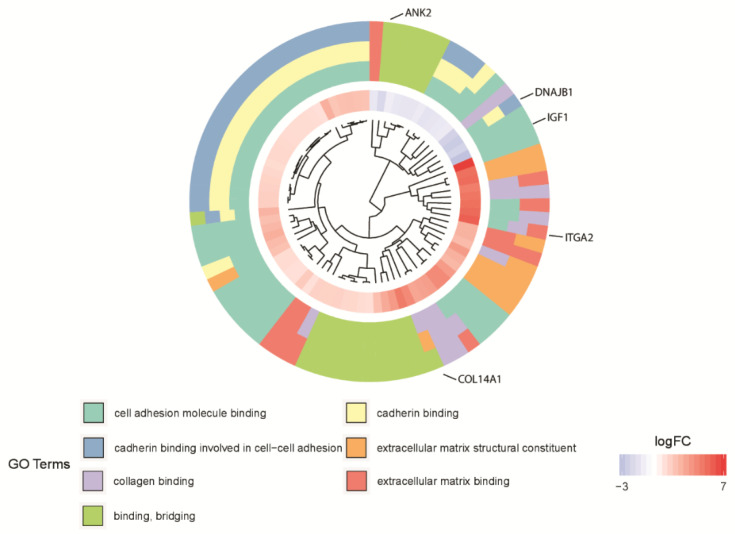
The dendrogram of the 81 differentially expressed genes involved in “extracellular matrix binding”, “extracellular matrix structural constituent”, “binding, bridging”, “cadherin binding”, “cell adhesion molecule binding”, “collagen binding” and “cadherin binding involved in cell-cell adhesion” GO MF terms. The DEGs were clustered based on their logFC values.

Subsequently, the interaction between the seven selected ontological groups of the 81 genes was evaluated. One of the most visually appealing ways of presenting such interaction is by using a dendrogram (Figure 4). Clusters contain functionally related genes based on their expression pattern. The middle circle represents a logarithm of fold change (logFC) of differentially expressed genes assigned to the studied GO terms. The GO terms are shown as the outer ring. The genes whose expression is downregulated form a small cluster (blue part of the middle circle—as can be seen for the gene IGF1), which is consistent with the previous observations. Clusters of the same color over the entire width of the outer circle represent genes that are unique for a specific GO term (as can be seen for the gene ANK2). Clusters of different colors on the cross-section of the outer circle show sets of genes that are likely to be functionally related (as can be seen for the genes DNAJB1, COL14A1, ITGA2). The dendrogram showed that 25 of the 81 genes belong simultaneously to three of the selected GO MF terms: “cadherin binding”, “cell adhesion molecule binding” and “cadherin binding involved in cell-cell adhesion”.

**Figure 5:**
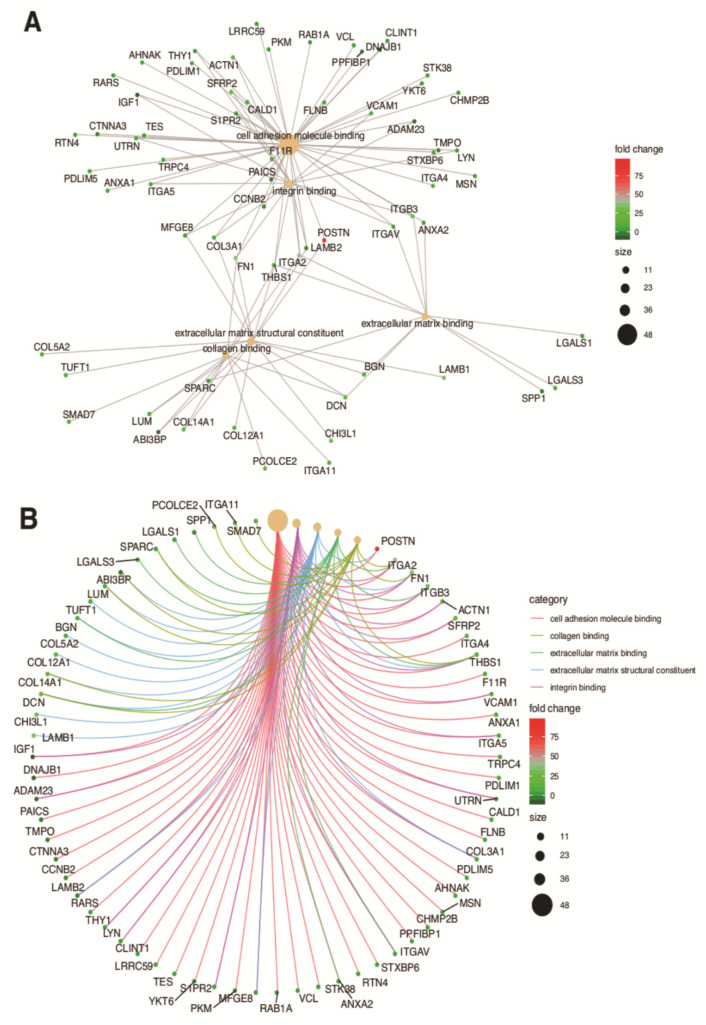
Analysis of enriched gene ontological groups involved in (**A**) cell adhesion molecule binding, collagen binding, extracellular matrix binding, extracellular matrix structural constituent, integrin binding. The network plots presenting the linkages of genes and GO MF terms; (**B**) cadherin and collagen binding and structuralization of extracellular matrix components. The network plots presenting the linkages of genes and GO MF terms.

**Figure 6:**
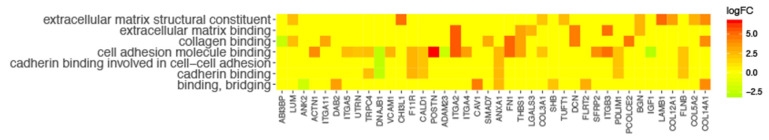
Heatmap presenting the relationship between genes and selected GO MF terms. Yellow color of tiles indicates the absence of logFC values.

In the gene ontology database, single genes may belong to many ontological terms. In our study, among 15 down-regulated genes, 5 of them belonged to two or more ontology groups. Of the 66 up-regulated genes, 33 showed belonging to more than one GO. Therefore, plots with visualization of fold change values and the relationship between genes and selected GO MF terms were employed to depict the data (Figure 5A,B). The relationship was also presented as a heatmap (Figure 6). Genes were selected on the basis of the logFC [2.5] (absolute value, i.e., >2.5 and <−2.5).

**Figure 7:**
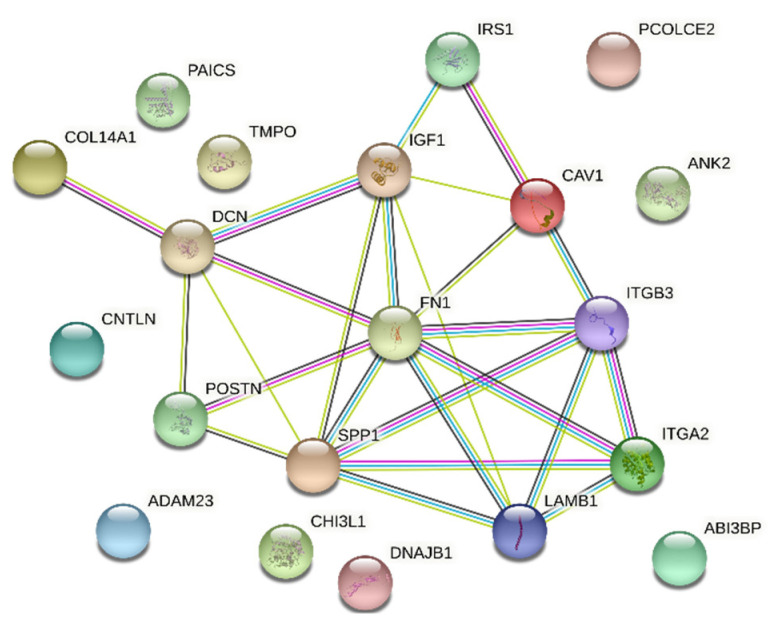
STRING-generated interaction network among 20 chosen differently expressed genes belonging to the “extracellular matrix binding”, “extracellular matrix structural constituent”, “binding, bridging”, “cadherin binding”, “cell adhesion molecule binding”, “collagen binding” and “cadherin binding involved in cell-cell adhesion” GO MF terms. The strength of the interaction score was reflected by the intensity of the edges.

**Figure 8:**
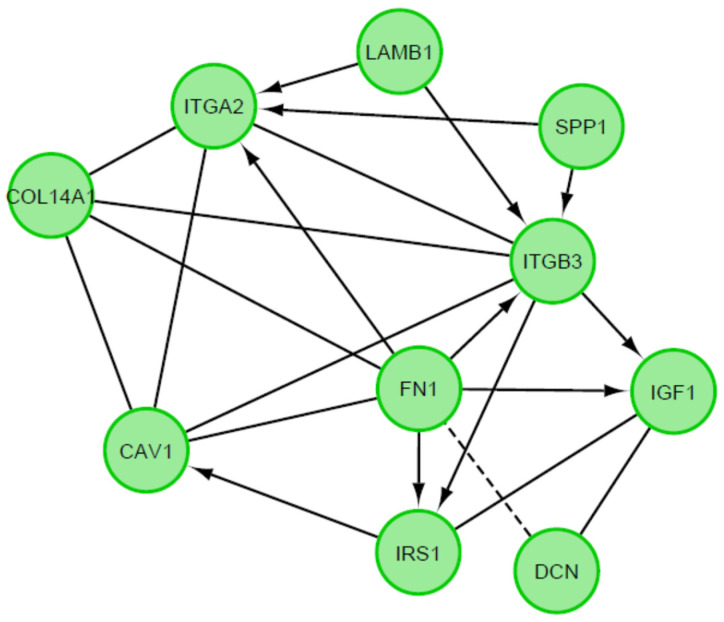
Functional interaction (FI) between 20 chosen differently expressed genes. In above figure “->“ stands for activating/catalyzing, “-” for FIs extracted from complexes or inputs, and “---” for predicted FIs.

A STRING interaction network was used to demonstrate the interaction between the 10 most up-regulated and the 10 most down-regulated genes analyzed, which can predict the relationship between the protein products of the genes described (Figure 7). In the last stage, the functional interactions between chosen genes with REACTOME FIViz app to Cytoscape 3.8.2 software were evaluated. The results were shown in Figure 8.

**Figure 9:**
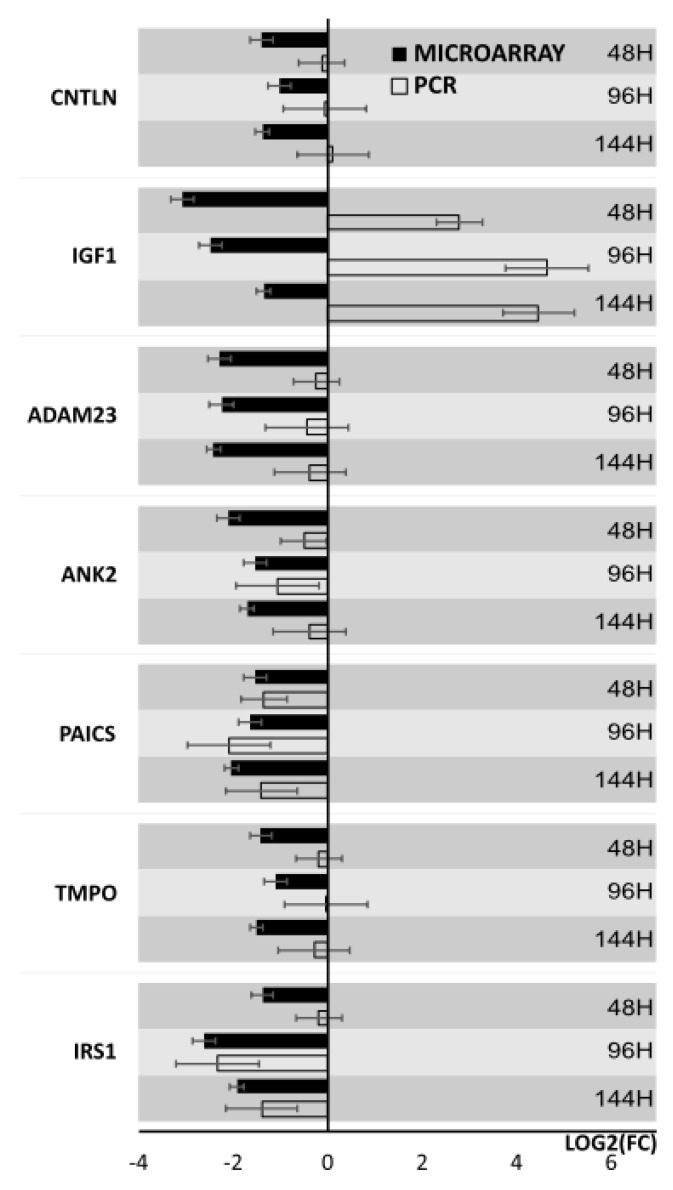
Bar graph showing validation results of microarrays of downregulated genes obtained by RT-qPCR.

**Figure 10:**
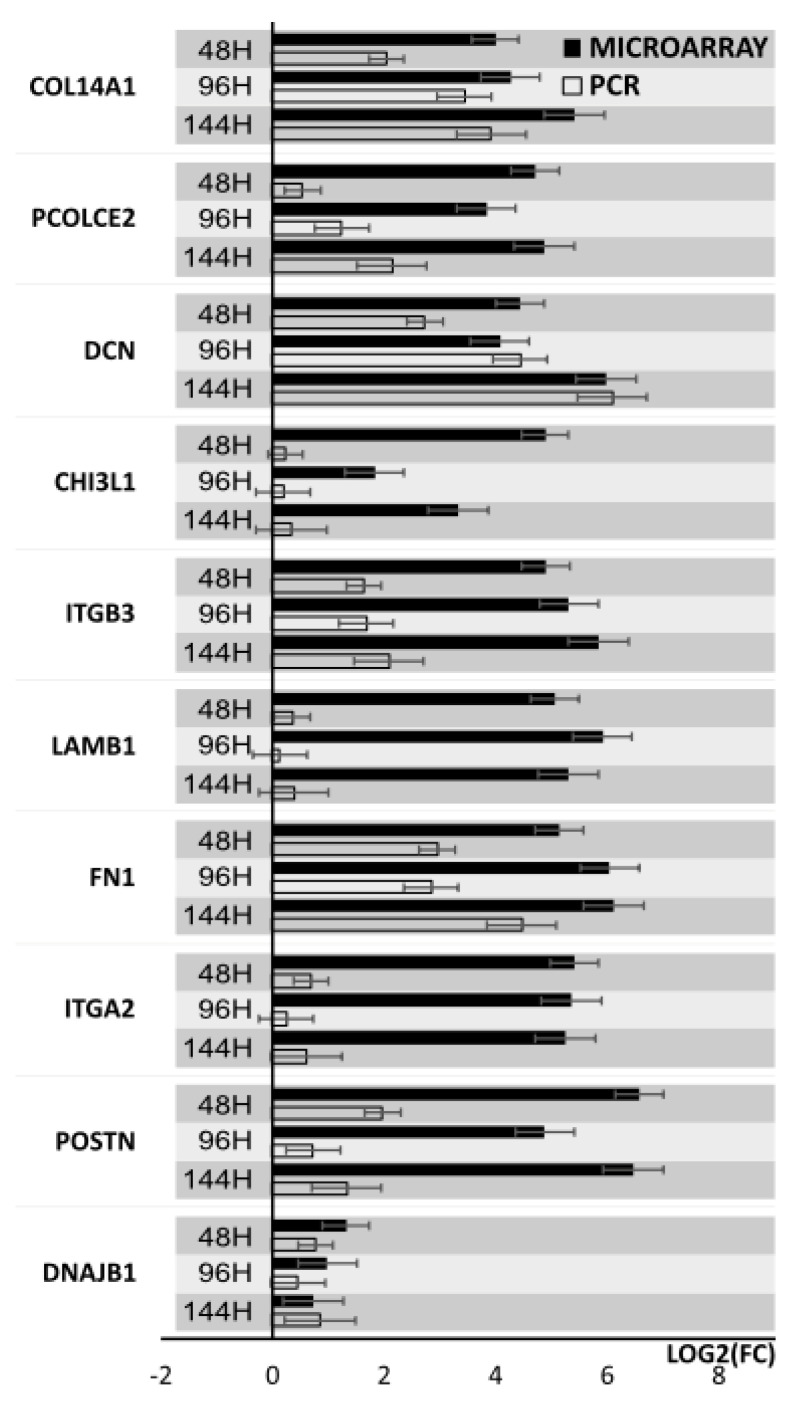
Bar graph showing validation results of microarrays of upregulated genes obtained by RT-qPCR.

Results from microarray expression were confirmed by quantitative RT-qPCR on porcine granulosa cells. These data sets were collected, compared, and presented as bar graphs (Figure 9 and Figure 10). All 20 selected genes were validated. For 3 downregulated genes via microarray analysis, the reaction conditions could not be established. Non-specific products were formed in the reaction. Only 7 downregulated genes have been presented in the graph (Figure 9). Moreover, the expression pattern for one gene (IGF1) was not confirmed. The expression direction of upregulated genes in all cases was confirmed (Figure 10). The direction of expression change was different on all days of culture intervals. The RT-qPCR result can be more representative because this method has greater quantitative precision, as opposed to the whole transcriptome analysis provided by microarrays.

## 4. Discussion

The contact of the cell with the external environment, including the ECM, is crucial for the continuity of the communication, the reception of stimuli, and the occurrence of basic processes such as migration, proliferation, differentiation, morphogenesis, as well as providing tissue homeostasis [25]. The high complexity of ECM composition, its modification in different tissues, and its activity prove that it is not only an extracellular spatial skeleton of tissues. The processes of cell–cell but also cell–ECM adhesion depend on many integrated networks of protein interactions as well as signaling pathways [52]. Considering the wide range of functions performed by the ECM, as well as its significance in cell function in vitro, it is very important to determine the changes in gene expression relative to in vivo conditions. Due to the relatively different function of cumulus cells (located in the closest proximity to the oocyte) and mural GCs, the gene expression profile between these subpopulations may differ. Thus, the changes that occur during in vitro culture can be significantly different, and knowledge of this transcriptomic profile will allow for further studies on GCs cultured in vitro. Further research, including in the field of assisted human and animal reproduction techniques, determination of mechanisms of ovarian pathology, or stemness, GCs use in regenerative medicine, may rely on the transcriptomic data. In this study, among 81 genes with statistically significant differential expression, 10 the most upregulated POSTN, ITGA2, FN1, LAMB1, ITGB3, CHI3L1, PCOLCE2, CAV1, DCN, COL14A1, and 10 the most down-regulated SPP1, IRS1, CNTLN, TMPO, PAICS, ANK2, ADAM23, ABI3BP, DNAJB1, IGF1 were selected for further analysis. 

**Figure A1:**
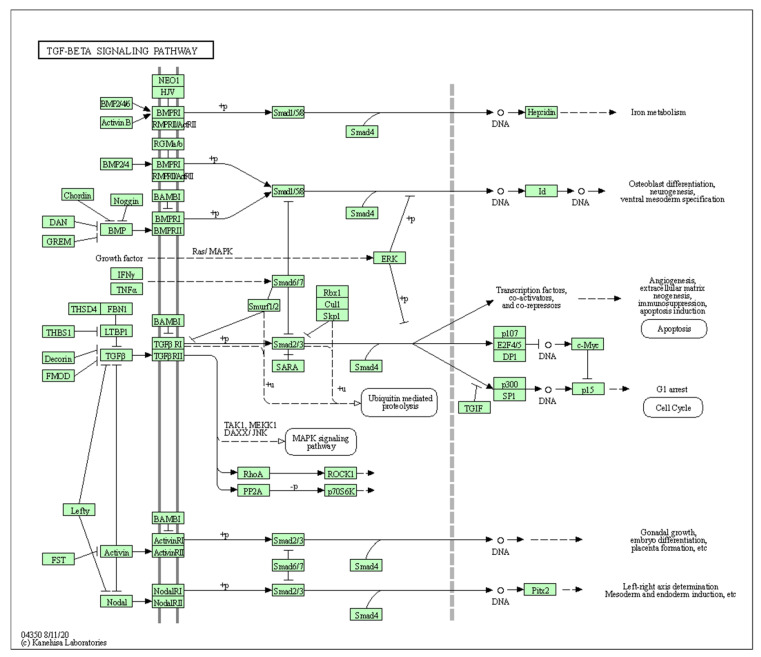
TGF-β signaling pathway, KEGG pathway. KEGG, Kyoto Encyclopedia of Genes and Genomes. Copyright permission obtained.

It has been shown that ECM components have a direct effect on biological processes within the ovary such as folliculogenesis, ovulation and steroidogenesis. Glycoproteins, which play an important role in ovulation are involved in signaling with other ECM components including proteins, integrins and growth factors, deserve special attention. These include, among others: DCN (decorin), FN1 (fibronectin) and POSTN (periostin). These genes showed a significant increase in expression during in vitro culture of GCs compared to the reference value. The description of increased expression of DCN in mural granulosa cells (MGCs) than cumulus granulosa cells (CGCs) may indicate involvement in ovulation. The effect of LH/hCG on the upregulation of this gene expression was also shown to be mediated through PKA, PKC, ERK/MEK, and PI3K pathways [53]. The role of DCN is to participate in cell proliferation, cell cycle regulation and apoptosis [54] and its expression has been described in human, goat, pig, and bovine granulosa cells [19,53,54,55]. Additionally, it may also be associated with cellular aging [56], folliculogenesis and ovulation [53]. Therefore, its role as a marker used in assisted reproduction biotechnologies is described [57]. On the other hand, the increased eCG-mediated DCN expression in goat granulosa may be disrupted by the tissue inhibitor of metalloproteinase 3 (TIMP3) present [58]. A role for DCN in steroidogenesis has also been suggested and demonstrated by resistin in vitro. Resistin is a protein for which one of the receptors is decorin, found in ovarian granulosa cells. It has been shown that the application of resistin can influence the regulation of steroidogenesis through Akt, MAPK, Stat-3, PPARγ, and NF-kB signaling pathways, leading to a decrease in estradiol and progesterone levels [59]. The DCN gene is associated with the TGF-β signaling pathway (Figure A1). This pathway indirectly affects cellular processes related to the cell cycle and apoptosis. 

**Figure A2:**
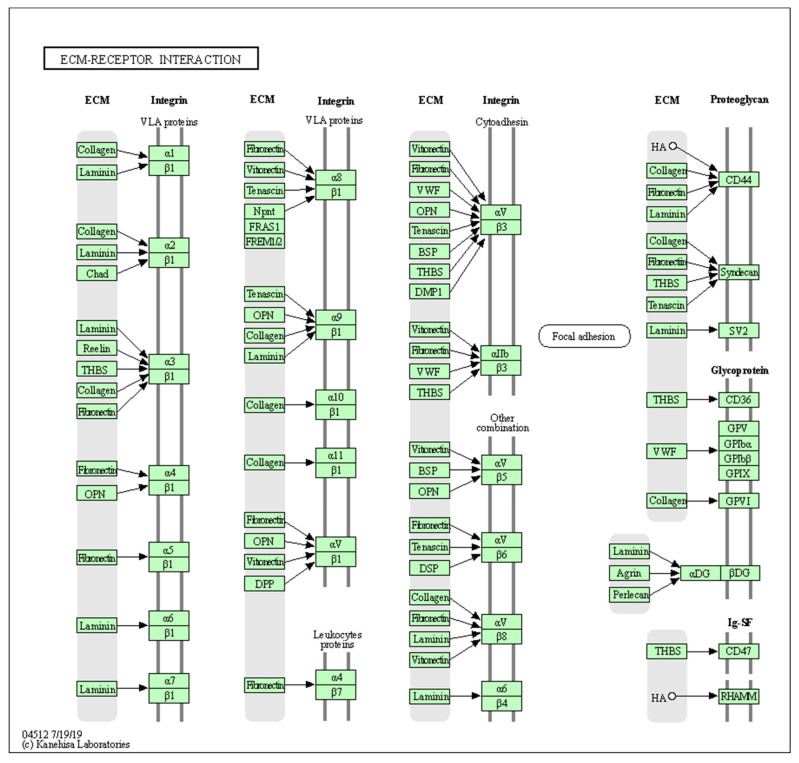
ECM-receptor interaction signaling pathway, KEGG pathway. KEGG, Kyoto Encyclopedia of Genes and Genomes. Copyright permission obtained.

**Figure A3:**
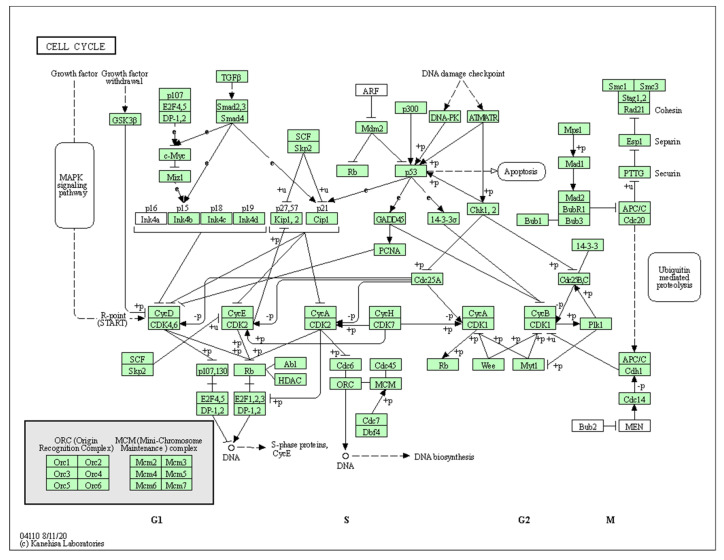
Cell cycle signaling pathway, KEGG pathway. KEGG, Kyoto Encyclopedia of Genes and Genomes. Copyright permission obtained.

**Figure A4:**
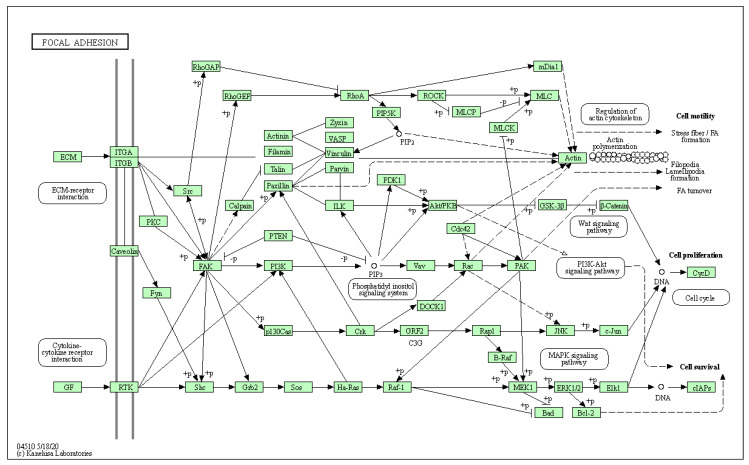
Focal adhesion signaling pathway, KEGG pathway. KEGG, Kyoto Encyclopedia of Genes and Genomes. Copyright permission obtained.

In turn, integrins are bound by various ECM components, including fibronectin (Figure A2). The type of this signaling interaction is referred to as input-output and involves the fibronectin receptor [60]. The fibronectin signaling pathway shows further association with MAPK/ERK, involved in the cell cycle (Figure A3). The FAK (focal adhesion kinase, Figure A4) signaling pathway engages FN1, an important component of the ECM, thereby mediating processes in the ovary, particularly ovulation, luteinization, and cumulus cell expansion [61]. Inhibition of the FAK pathway results in the arrest of ovulation, demonstrating the crucial role of the ECM in this process [62]. Fibronectin was shown to be involved in ovulation and luteinization of GCs, as it enhanced progesterone production in vitro, compared to collagen, which promoted estrogen synthesis [63]. Additionally, an increase in the expression of this gene in GCs of beef cattle after super-stimulation was demonstrated [64]. 

It is worth considering that the expression of this gene in the follicular fluid of lambs is lower than in sheep [65] indicating that the expression may depend on various factors related to the physiological status of the animal. It has been described that under the stimulation of hCG in bovine granulosa cells the expression of the POSTN gene increases and its role in fibrous tissue remodeling may induce ovulation [66]. This gene has also been shown to influence cell adhesion, migration, proliferation, differentiation, and survival [67]. Confirmation of the role of this gene in the aforementioned processes may be provided by the relationship between elevated POSTN levels and PCOS in humans [9]. The role of FN1 in PCOS is associated with effects on vasculature development within the ovary, which significantly affects folliculogenesis and corpus luteum formation [10].

Another glycoprotein showing roles in ECM remodeling is CHI3L1 (chitinase-3-like protein 1 CHI3L1) gene, which also showed up-regulation during conducted culture. Its high expression level is an indicator of poor prognosis for ovarian cancer patients [68] and can be used as a goal for targeted therapy [69]. Summarizing the described glycoproteins and their role in ovarian processes, it should be pointed out that they are mainly responsible for ovulation. Although additionally their role in steroidogenesis has been demonstrated. It is also important that the above ECM elements are associated with the occurrence of PCOS. These data and our results show that not only the glycoproteins themselves but also their association with integrins and their participation in signaling pathways are important in the assessment of their role in this disorder. Demonstration of the expression levels of these genes in the context of their interactions may be a basis for studies related to the therapy of, among others, PCOS and their strong upregulation within GCs and association with ovulation may be used in reproductive biotechnologies based on in vitro studies. This suggests that further research is needed in this direction, but gives hope that glycoproteins may be a target for therapy and use in assisted reproductive techniques.

**Figure A5:**
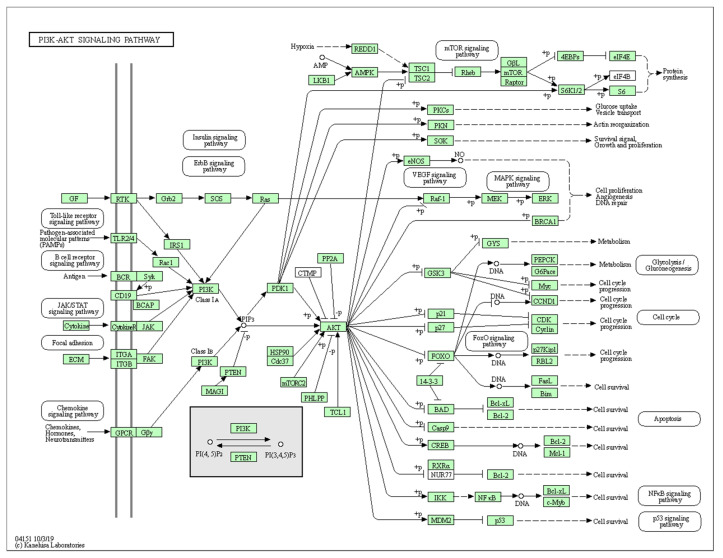
PI3K-AKT signaling pathway, KEGG pathway. KEGG, Kyoto Encyclopedia of Genes and Genomes. Copyright permission obtained.

Not only the ECM glycoproteins mentioned above are involved in these processes within the ovary. CAV1 (caveolin-1) protein is strictly responsible for the composition of the extracellular matrix. It is involved in the formation of exosomes and sorting of protein components of the extracellular matrix as well as their deposition [70]. Thus, it leads to changes in ECM composition and, consequently, its function. CAV1 gene expression in bovine granulosa cells and its role in the process of ovulation and luteinization have been demonstrated [71]. Given the role of the CAV-1 gene in extracellular matrix formation, it has been shown that in vitro culture its expression is affected by anethole. It was observed that anethole, as a natural substance with antioxidant properties, influences the maturation of goat ovarian follicles [72]. It has also been shown that downregulation of CAV-1 gene expression in female ovaries pathway affects the Notch2 signaling and causes a decrease in Leucine-rich repeat containing G protein-coupled receptor 5 (Lgr5) leading to POI (premature ovarian insufficiency) [11]. Lgr5 protein affects cell proliferation, differentiation and is also a biomarker of adult stem cells [73]. Moreover, the IRS1 gene (insulin receptor substrate 1) protein, which showed downregulated expression in our study compared with the reference value, is associated with the mitogen-activated protein kinase (MAPK)/extracellular signal-regulated kinase (ERK) pathway and also PI3K/AKT (Figure A5) [74,75]. IRS1 affects cell metabolism regulation, survival, and apoptosis [76]. The association of its overexpression with GCs proliferation was demonstrated in PCOS (polycystic ovary syndrome) study, where it activated the MAPK/ERK pathway and promoted the disorder [77]. The MAPK/ERK pathway is also associated with ovulation, as it is completely inhibited in mice with MAPK1/3 deletion in GCs [78].

**Figure A6:**
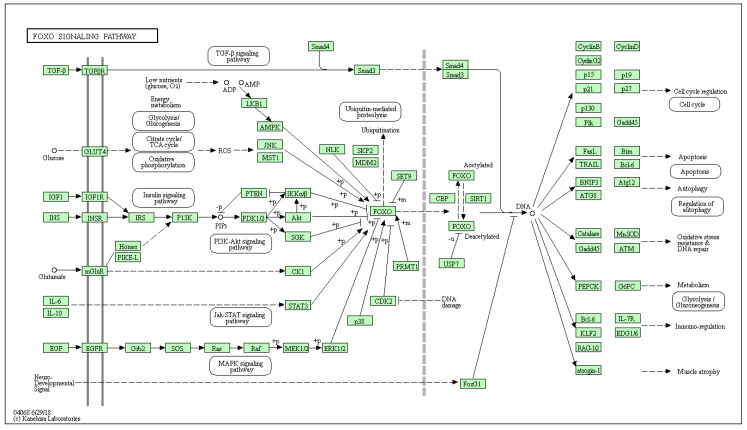
FOXO signaling pathway, KEGG pathway. KEGG, Kyoto Encyclopedia of Genes and Genomes. Copyright permission obtained.

**Figure A7:**
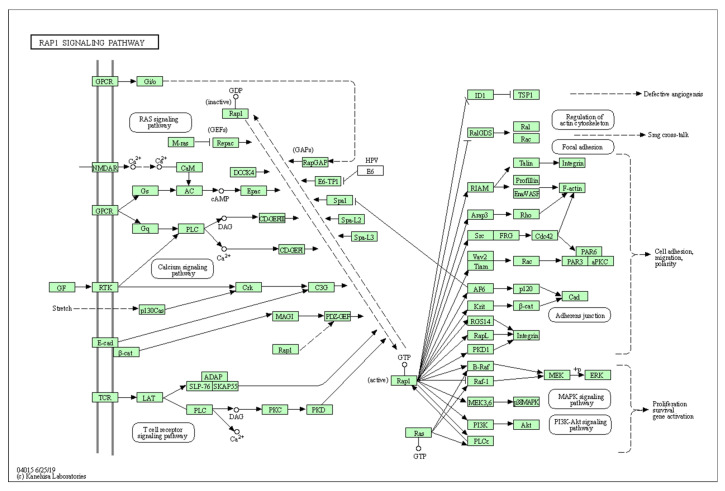
RAP1 signaling pathway, KEGG pathway. KEGG, Kyoto Encyclopedia of Genes and Genomes. Copyright permission obtained.

A group of widely distributed and important proteins are integrins, adhesion molecules, as transmembrane proteins are composed of α and β components [79]. ITGA is associated with ECM formation, ITGB plays one of the key roles in regulating intracellular signaling cascades (including FAK, AKT—Figure A4 and Figure A5). With reference to carcinogenesis, in particular ovarian cancer, a role for ITGA2 in spheroid formation is described, thus promoting metastasis in the peritoneal cavity [80,81,82]. The PI3K/AKT pathway is also closely related to the FOXO family proteins (Figure A6), a member of the transcription factor class. The upregulated ITGA2 gene affects AKT phosphorylation [83], making it closely related to the FAK signaling pathways. Integrins bind to focal adhesion components to identify modifications in the ECM to keep the shape or reshape the cell [84,85]. Cadherin–integrin interactions play an important role, involving among others the Rap1 pathway [60], associated with GTP (Figure A7). Rap1 is involved in the regulation of integrin activity downstream of cadherins [86] and maintains cadherin connections [87]. ITGA2, ITGB3 genes play an important role in many signaling pathways. The upregulation of these genes has been previously described in porcine granulosa cells, and it was associated with their angiogenesis [55], development, morphogenesis [88], adhesion, tissue development [89], and in the case of ITGB3 and FN1 also with the process of apoptosis [56]. These three genes belong to ontology groups closely related to cell adhesion, formation, and binding of extracellular matrix components confirming the role of the ECM in the processes previously described related to granulosa cell proliferation [55,88,89,90]. Decreased expression of the genes mentioned above affects ECM and cell adhesion within the ovary, affecting the possibility of diseases within the ovary, including PCOS (polycystic ovary syndrome) in humans. It is important to consider the role of ECM in folliculogenesis and ovulation [91]. Furthermore, in the current study, the down-regulated gene ABI3BP (ABI family member 3 binding protein), encoding an extracellular matrix protein that binds to integrins has been demonstrated. Studies on MSCs have shown that their activity is essential for osteogenic and adipogenic differentiation [92]. Through association with the fibronectin III domain, it promotes cell attachment and with the ECM [93]. 

One of the main components of lamina basalis is laminin, which binds to collagen. Increased expression of the laminin subunit beta 1 (LAMB1) gene, which belonged to the ontology group (GO: ECM structural constituent) is illustrated in Figure 1. The LAMB-1 gene has been described in ovarian follicles at different developmental stages and also in the corpus luteum in mice [18]. In addition, laminin protein can influence the shape and proliferation of GCs and also indirectly affect estradiol secretion in the antral follicles [63]. Thus highlighting the role of laminin, a basic component of lamina basalis in human folliculogenesis [94].

Another important component of the lamina basalis is collagen, which during the development of the ovarian follicle may change its conformation, amount, and proportion of isoforms [19]. In the present study, increased expression of PCOLCE2 (procollagen C-endopeptidase enhancer 2) and COL14A1 (collagen type XIV alpha chain) genes, which among others belong to the “collagen binding” ontology group was observed. Thus, up-regulation of the aforementioned genes indicates active modeling of ECM composition in primary in vitro culture of porcine GCs. Other ongoing in vitro studies have demonstrated increased expression of collagens (*COL1A2*, *COL3A1*, *COL5A2*, *COL12A1*, *COL15A1*, *COL6A3*) in the theca interna of the bovine ovarian follicle, leading to their consideration as cellular markers of this structure [95].

IGF1 (insulin-like growth factor 1) is one of the described regulators of folliculogenesis and oogenesis and is also involved in cell proliferation. Expression of this gene was the most strongly reduced during in vitro culture of porcine GCs relative to the reference value. The number of its receptors in the ovary changes depending on the presence of the corpus luteum and is therefore dependent on steroid hormones [96]. In turn, down-regulation of IGF1 is associated with apoptotic processes [97], and it has additionally been shown to increase apoptotic gene regulation through activation of PI3K/AKT (Figure A4) in bovine granulosa cells [98]. Another gene with reduced expression is SPP1 (secreted phosphoprotein 1), which was shown in bovine GCs where its expression level in the largest follicles (>10.7 mm) was lower than in smaller follicles (>7.8 mm) [99]. This demonstrates that the expression profile of follicle stage-specific genes is altered and is associated with development or atresia. This gene is otherwise known as osteopontin (OPN) and is associated with immune processes, particularly during ovarian follicular atresia [100]. It also plays an important role in wound healing processes, as it is responsible for cell adhesion to the ECM through the integrin-binding sequence [101]. 

The down-regulated PAICS gene (phosphoribosylaminoimidazole carboxylase and phosphoribosylaminoimidazolesuccinocarboxamide synthase) encodes an enzyme that catalyzes steps 6 and 7 of the de novo purine biosynthesis pathway [102] and was indicated in the corpus luteum in cattle. Its expression level was shown to be higher in the ovary than in skeletal muscle, indicating that these tissues require high levels of purines for the normal proliferation of GCs [103].

Both physical and functional interactions between particular genes are confirmed by STRING interaction (Figure 7). Interactions are mainly formed by genes closely related to extracellular matrix formation. These connections occur mostly between upregulated genes, although they also occur between downregulated genes IGF1, IRS1, SPP1. The gene showing the highest number of interactions was FN1, which by binding to genes encoding extracellular matrix glycoproteins confirms its participation in transmitting intercellular signaling. In addition, the strongly expressed interaction of FN1 to integrins ITGA2 and ITGB3 (and between) confirms the activity of these genes and their influence on the numerous signaling pathways described above. Also noteworthy is the connection between the DCN and IGF1 genes and between SPP1 and ITGB3. These genes, among others, are associated with apoptosis processes and may have a strong influence on changes within granulosa cells leading to ovulation, which may confirm their involvement in this process. Other genes that do not show direct interaction on STRING interaction may show it indirectly, therefore, their function in ovarian processes cannot be excluded. 

## 5. Conclusions

The incompletely understood molecular basis of processes within the ovarian follicle leading to ovulation and luteinization requires precise determination of the gene expression profile within, among others, granulosa cells. Therefore, the results can be broadly applied not only to understand the physiology of the ovary but also to study pathological processes and assisted reproduction techniques. In addition, compared to in vivo studies, in vitro methods are less expensive, easier to perform, and do not require the approval of ethical committees if the material used in the study comes from commercially slaughtered animals. The expression profile of genes in porcine granulosa cells cultured in vitro, associated with cadherin and collagen binding and structuralization of extracellular matrix (ECM) components were carefully evaluated, and the culture of GCs in vitro showed increased expression of genes highly responsible for extracellular matrix (ECM) formation. ECM is involved in the regulation of many processes within the ovarian follicle, including its development, maturation, ovulation, and corpus luteum formation. The pig has been used as a model in human medicine, so the results of this study may be valuable in determining pathological mechanisms within the ovary, including finding targets for clinical therapy in humans. Of special attention is the description of the expression levels of genes such as POSTN, CHI3L1, CAV-1, IRS1, DCN, which according to literature data are associated with diseases such as POI, PCOS, and ovarian cancer. The current and previously demonstrated increased expression of POSTN, FN1, ITGB3, ITGA2, LAMB1, DCN genes in granulosa cells, associated with the processes of growth, proliferation, maturation and differentiation, may become potential new molecular markers of proliferation of these cells in vitro what can be valuable in assisted animal reproduction techniques. Additionally, the described expression profile of genes related to extracellular matrix structuralization, which plays an important role in stem cell differentiation and regeneration processes, may provide important knowledge in this aspect. Signaling pathways and interactions associated with the described genes demonstrate the multidirectional role of the ECM in both spatial structure formation, intercellular signaling describing even more fully the role of selected genes.

## Figures and Tables

**Table 1:** Oligonucleotide sequences of primers used for RT-qPCR analysis.

Gene	Primer Sequence (5′-3′)		Product Size (bp)
IRS1	F	CCTAGCACCAACAGGACTCA	239
	R	GAAGAGATGAAACCGCCGTC	
TMPO	F	GCTCAGTGGAAAGTCAGCAG	241
	R	CCTGTCAATTTGCTGCCACT	
PAICS	F	AGTCATGCTACACAGGCCAT	235
	R	TTACCATCTGCAGCCCTTCA	
ANK2	F	TTGTAACGGAGGAGGTCACC	222
	R	AACGCAGGTAGTTCATCCCA	
ADAM23	F	AGCAGCTCAATACCAGGGTT	235
	R	TTCACACCAACTCCCCTTGT	
IGF1	F	TTCTACTTGGCCCTGTGCTT	222
	R	CTCCAGCCTCCTCAGATCAC	
CNTLN	F	ACCTCAACCCATAAAGCCCA	186
	R	TGTGGCAAAAGGAAGCTGTC	
DNAJB1	F	AGGACCATACCCGTTGTGTT	167
	R	AGGACGGTTCTTGAGGTCTG	
POSTN	F	ATTGACCGTGTCCTCACACA	212
	R	GCCACTTTGTCTCCCATGAT	
ITGA2	F	CATGCCAGATCCCTTCATCT	153
	R	CGCTTAAGGCTTGGAAACTG	
FN1	F	TGAGCCTGAAGAGACCTGCT	113
	R	CAGCTCCAATGCAGGTACAG	
LAMB1	F	CTTCACCACCTTGGACCACT	216
	R	AGCTGTGGCTCATAGCGAAT	
ITGB3	F	GGCTTCAAAGACAGCCTCAC	175
	R	AGTCCTTTTCCGAGCACTCA	
CHI3L1	F	GGATGCAAGTTCCGACAGAT	202
	R	GAGGATCCCTTTCTCCTTGG	
DCN	F	CTCTCTGGCCAACACTCCTC	155
	R	GCGGGCAGAAGTCATTAGAG	
PCOLCE2	F	TGTAAACGGACTGGGACTCC	184
	R	CGATGACCTTGGCACTCATG	
COL14A1	F	AGTTCCAGCCCAGCAATACT	229
	R	ATCGTCCAGTACAGCCAACA	

**Table 2:** The 10 most significantly upregulated and 10 most significantly downregulated genes involved in cadherin and collagen binding and structuralization of extracellular matrix components.

Gene Symbol	Gene Name	Fold Change	Adj. p. val.
POSTN	periostin, osteoblast specific factor	95.2	3.5 × 10^7^
ITGA2	Integrin, alpha 2 (CD49B, alpha 2 subunit of VLA -2 receptor)	42.7	3.5 × 10^7^
FN1	Fibronectin 1	35.4	3.6 × 10^7^
LAMB1	Laminin, beta 1	33.3	3.5 × 10^7^
ITGB3	Integrin, beta 3 (platelet glycoprotein IIIa, antigen CD61)	29.9	3.5 × 10^7^
CHI3L1	Chitinase 3-like 1 (cartilage glycoprotein-39)	29.6	1.4 × 10^5^
PCOLCE2	Procollagen C-endopeptidase enhancer 2	26.2	4.6 × 10^7^
CAV1	Caveolin 1, caveolae protein, 22kDa	22.3	4.3 × 10^7^
DCN	decorin	21.6	4.4 × 10^7^
COL14A1	Collagen, type XIV, alpha 1	16.0	5.3 × 10^7^
SPP1	Secreted phosphoprotein 1	−2.5	1.4 × 10^2^
IRS1	Insulin receptor substrate 1	−2.6	1.9 × 10^4^
CNTLN	Centlein, centrosmal protein	−2.6	5.1 × 10^4^
TMPO	thymopoietin	−2.7	1.3 × 10^4^
PAICS	Phosphoribosylaminoimidazole carboxylase, phosphoribosylaminoimidazole succinocarboxamide synthetase	−2.9	8.0 × 10^5^
ANK2	Ankyrin 2	−4.3	2.1 × 10^5^
ADAM23	ADAM metalloptidase domain 23	−4.9	1.0 × 10^5^
ABI3BP	ABI family, member 3 (NESH) binding protein	−5.3	2.1 × 10^5^
DNAJB1	DnaJ (Hsp40) homolog, subfamily B, member 1	−6.4	2.3 × 10^6^
IGF1	Insulin-like growth factor 1 (somatomedin C)	−8.3	1.0 × 10^4^

## Data Availability

Not applicable.
